# Supervised machine learning applied in nursing notes for identifying the need of childhood cancer patients for psychosocial support

**DOI:** 10.3389/fdgth.2025.1585309

**Published:** 2025-08-07

**Authors:** Akseli Reunamo, Hans Moen, Sanna Salanterä, Päivi M. Lähteenmäki

**Affiliations:** ^1^ Department of Computing, University of Turku, Turku, Finland; ^2^ Department of Computer Science, Aalto University, Espoo, Finland; ^3^ Department of Nursing Science, University of Turku, Turku, Finland; ^4^ Nursing Administration, Turku University Hospital, Turku, Finland; ^5^ Department of Pediatric and Adolescent Medicine, Turku University Hospital, FICAN-WEST and University of Turku, Turku, Finland

**Keywords:** cancer, nursing notes, machine learning, electronic health records, psychosocial support systems, late effects

## Abstract

**Introduction:**

Childhood cancer survivors have a higher risk of mental health and adaptive problems compared with their siblings, for example. Assessing the need for psychosocial support is essential for prevention. This project aims to investigate the use of supervised machine learning in the form of text classification in identifying childhood cancer patients needing psychosocial support from nursing notes when at least 1 year had passed from their cancer diagnosis.

**Methods:**

We evaluated three well-known machine learning–based models to recognize patients who had outpatient clinic reservations in the mental health–related care units from free-text nursing notes of 1,672 patients. For model training, the patients were children diagnosed with diabetes mellitus or cancer, while evaluation of the model was done on the patients diagnosed with cancer. A stratified fivefold nested cross-validation was used. We designed this as a binary classification task, with the following labels: no support (0) or psychosocial support (1) was needed. Patients with the latter label were identified by having an outpatient appointment reservation in a mental health–related care unit at least 1 year after their primary diagnosis.

**Results:**

The random forest classification model trained on both cancer and diabetes patients performed best for the cancer patient population in three-times repeated nested cross-validation with 0.798 mean area under the receiver operating characteristics curve and was better with 99% probability (credibility interval −0.2840 to −0.0422) than the neural network–based model using only cancer patients in training when comparing all classifiers pairwise by using the Bayesian correlated t-test.

**Conclusions:**

Using machine learning to predict childhood cancer patients needing psychosocial support was possible using nursing notes with a good area under the receiver operating characteristics curve. The reported experiment indicates that machine learning may assist in identifying patients likely to need mental health–related support later in life.

## 1 Introduction

Because of improved cancer treatments, more than 80% of European children and adolescents who develop cancer will survive for more than 5 years. There are over 300,000 survivors of childhood and adolescent cancer across Europe, and roughly 5,000 adult survivors in Finland. This number is constantly growing (1). However, cancer treatments are physically and mentally heavy and burdensome, and due to long-term effects, survivors require closer health monitoring than the average population (2). A growing body of literature during the last four decades has shown that lifelong survivorship care is needed for most survivors. International standards for psychosocial support in different treatment phases have been published (3). Still, most often, the focus in practice, at least in Finland, is on early postdiagnosis and patients receiving palliative care. Survivorship care is complex (4). Survivors may experience psychological distress and have impaired health, yet often portray a positive perspective on their lives (5). However, later in the adult lives of survivors, there are indications that some survivors are at a higher risk of anxiety and depression when compared with their siblings (6). In addition, oncologists responsible for follow-up of survivors may have problems recognizing patients in distress (7). Although the absolute risk of post-traumatic stress symptoms (PTSS) is low (8), in one recent report, long-term survivors with PTSS (14.5%) reported more impairment in mental and physical health–related quality of life compared with other childhood cancer survivors (9). Furthermore, young adult survivors are at risk of delayed psychosexual development and non-independent living (6).

To ensure a good quality of life after childhood cancer, targeted follow-up interventions and support strategies also addressing late mental effects and social difficulties are important (10). To provide appropriate care, a three-level model to guide decisions about the intensity and frequency of follow-up care was published two decades ago (11, 12). This risk-stratified approach is based on cancer diagnosis and treatments, specifically cumulative exposures. However, the model is missing the psychosocial profile. Traditionally, the psychosocial condition of childhood cancer patients (CCPs) has been evaluated by questionnaires (13) or by performing repetitive assessments to find disposition for small or high risk of maladjustment (14). Separate evaluations via questionnaires are resource-consuming, especially if computer-assisted tools linked to electronic medical records are not available. To the best of our knowledge, there is no validated tool that fits all age groups. Thus, the international standards for psychosocial support (3) after the treatment phase are often not fulfilled.

In Finland, clinicians have started implementing survivorship care based on the existing guidelines (15). Still, there is a need for innovations, such as automated tools, to assess the risk of late effects for each individual and to integrate the psychosocial factors into the mentioned risk calculation model (11, 12). We have published the first results of using electronic journal data via a hospital data lake for determining the above-mentioned late-effect risk group (16). Recent reports emphasize the importance of integrating psychosocial risk screening into clinical care (17, 18) and continuous surveillance of mental health problems (19). The present study reports our initial approach of using text classification models to predict the psychosocial state of health of CCPs during the postdiagnosis and follow-up phase of cancer treatment at the pediatric hematology-oncology clinic.

Nurses write patient condition observations into patient charts in a narrative format. This text is often informative and provides a clear picture of the patient's physical and psychosocial condition. These texts are mainly used for current hospital stays of patients. Natural language processing (NLP) allows us to use and analyze these texts for further purposes. Natural language text is among the most complex data types for storing and managing information. Thanks to continuous advancements in machine learning (ML) and NLP, computers can perform increasingly complex tasks on this data type. Applying text classification to nursing notes from patient electronic health records (EHRs) will enable computer-assisted detection, extraction, and classification of documented information, which may ultimately improve patient treatment quality, efficiency, and outcomes.

Recent research in ML and NLP has explored various approaches to identifying the need for psychosocial support in the context of cancer treatment and survivorship. Huang et al. (20) worked with structured data (categorical variables) and employed a hybrid feature selection and classification approach that included the use of deep learning neural networks (NNs) and support vector machines (SVMs) to predict long-term behavioral outcome risks in pediatric cancer survivors. Sheikhalishahi et al. (21) conducted a comprehensive review of ML methods/models applied to chronic disease prediction using unstructured clinical text. Common methods include logistic regression (LR), random forest (RF), NNs, and SVMs. Zeinali et al. (22) reviewed the classifiers used for symptom prediction in people with cancer. The data used in these studies seem to be primarily structured data. Common classification methods used include LR, RF, NNs, and combinatory approaches. The evaluation approaches typically include the use of cross-validation. McRoy et al. (23) utilized supervised methods, including naïve Bayes and random forests, for classifying health forum posts to assess unmet informational needs among breast cancer survivors. Lu et al. (24) analyzed methods/models for short-term mortality forecasting (≤1 year) from structured health record data, reporting the performance of models such as XGBoost and recurrent neural networks (RNNs). Masukawa et al. (25) focused on detecting social and spiritual distress in terminally ill cancer patients. Focusing on the unstructured free-text data in patient EHRs, they applied a bag-of-words (BoW) feature extraction and combined it with a range of different classifiers, especially LR, RF, SVM, and ensemble classifiers. More recently, Nunez et al. (26) applied Transformer-based models to initial oncology consultation notes to predict which cancer patients would later access psychiatric or counseling services. In a different approach, Korach et al. (27) used latent Dirichlet allocation to extract latent topics in nursing notes that predict deterioration, to enable timely interventions ultimately. Moen et al. (28) explored some approaches for detecting mentions of pain and acute confusion in free-text clinical notes. This included mention-level named entity recognition (NER) using conditional random fields (CRFs) and sentence-level classification using RNNs. Liukas et al. (29) annotated psychosocial factors in nursing notes associated with postoperative persistent pain, resulting in the identification of words and mentions that are important for enabling automated prediction and decision support.

In this research, we explore three text classification models to automate the detection of CCPs who may need additional support. With early detection of signs of distress, supportive measures could be started during childhood and adolescence and, thus, possibly help prevent psychosocial problems in adulthood. More specifically, we used a fully connected NN, RF, and an LR model. For the training phase of the models, we included patients with diabetes mellitus type I in addition to CCPs, because at our hospital, these patients are followed up until adulthood in the pediatric unit, and after the initial diagnostic phase, there is no systematic mental health screening used for them either. The goal of inclusion was to assess whether it increases the performance when predicting CCPs.

## 2 Materials and methods

All analyses were performed in a secure computing environment, with Intel® Xeon® Platinum 8160 CPU and NVIDIA Tesla V100 SXM2 32GB GPU. All scripts and software packages used in the analysis can be found on GitHub (30). Model development and reporting followed the Transparent Reporting of a multivariable prediction model for Individual Prognosis Or Diagnosis (TRIPOD) checklist for Prediction Model Development and Validation (31).

### 2.1 Ethics approval and consent to participate

This current study is based on EHR data aggregated into a data lake of one institution, Turku University Hospital. In Finland, the pure registry-based studies conducted according to the Act on the Secondary Use of Health and Social Data (26.4.2019/552, Finlex) are not evaluated by any Committee on Ethics but by the National Review Board at FINDATA. However, if all the data are gathered within one institution only, then permission to use these data is obtained from the institutional review board and not from FINDATA. The Act on the Secondary Use of Health and Social Data (26.4.2019/552, Finlex) describes the details, and as registry-based studies come under this category, no informed consent is requested either. Furthermore, all the data must be handled in a secure operating environment provided by the institution in question. The research plan was approved in 2019 by the Clinical Research Board of the Turku University Hospital. The permission number is T387/2019-1.

All the analyses were performed according to the approved study protocol following the relevant guidelines and regulations. The secure operating environment was provided by Auria Clinical Informatics on behalf of Turku University Hospital.

### 2.2 Data

**Figure 1:**
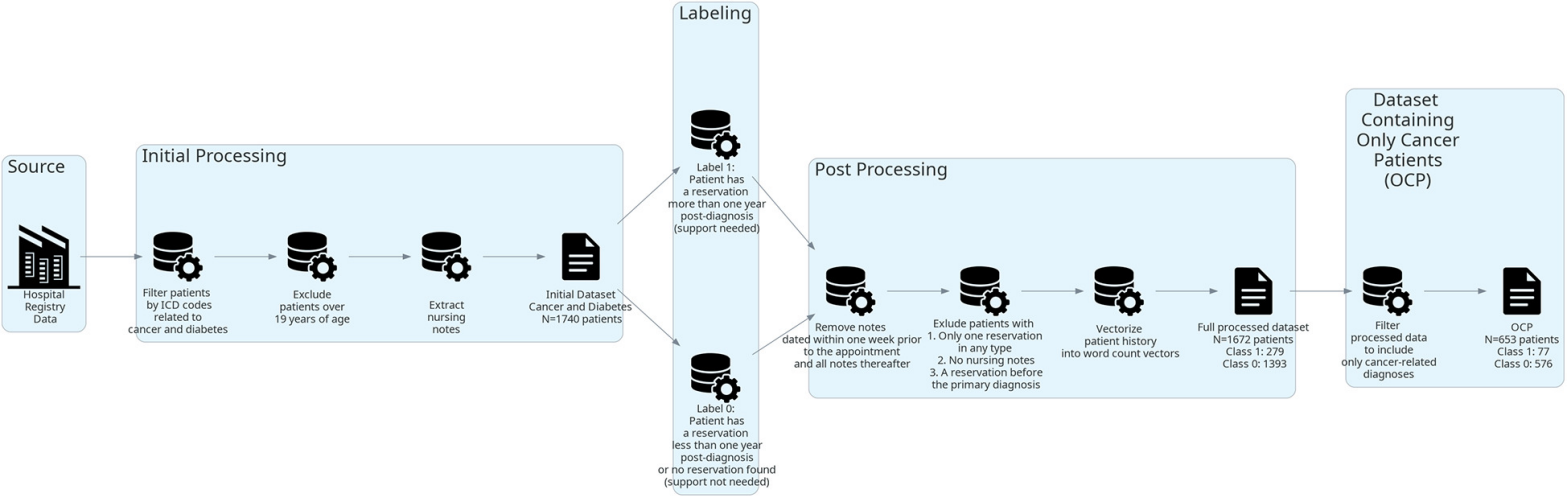
Flow diagram of data processing.

Data processing is described visually in Figure 1. The dataset consists of free-text nursing notes from patient EHRs and was obtained from a Finnish university hospital as retrospective registry data. In total, the whole data consisted of 185,525 nursing notes, from 1 January 2005 to 5 November 2020, of 1,740 different patients who had been diagnosed with cancer (C00.0–C97), tumor disease (D33.0–D33.9, D35.30, D46.0–D46.9, D47.0–D47.9), or diabetes mellitus type I (E10.00–E10.9) before the age of 19 years without any selection of genders. One nursing note refers to a text documented by a healthcare professional based on contact with a patient during daily care in a ward or at an outpatient visit. These nursing notes consist of free-text narratives that may contain typing errors, informal language, and medical jargon. Patients with diabetes mellitus were included in the data to increase the training sample size with patients who have a chronic disease that requires lifelong regular health status controls and psychosocial support. In addition to a set containing both diabetes and cancer patients, a separate dataset containing only cancer patients (OCP) was formed. If the patient had both diabetes and cancer-related diagnoses, cancer was set as the primary diagnosis. All patients who had an outpatient appointment booked at a mental health–related care unit after a year had passed from their primary diagnosis were labeled as “psychosocial support needed” (1) and others as “no need for support” (0). Nursing notes produced within 1 week before the booked appointment or later were removed from both labels.

After labeling, the whole dataset contained 1,672 patients. Twenty-four patients were excluded from the dataset due to having only one reservation of any type, two patients due to having no nursing notes, three due to having only one nursing note, and 39 due to having mental health–related contact before the primary diagnosis. Class 0 contained 1,393 patients (576 with cancer-related diagnoses and 817 with diabetes) and class 1 contained 279 patients (77 with cancer-related diagnoses and 202 with diabetes). The mean token count per care history was 9,857, with a maximum of 153,535 tokens and a minimum of 29 tokens. In this case, a token is an individual syntactic unit, for example, a word.

The OCP dataset contained 653 patients. Fourteen patients were excluded due to having only one reservation in any type, 3 due to having only one nursing note entry, and 16 having mental health–related contact before their cancer diagnosis. Class 0 contained 576 patients, and class 1 contained 77 patients. The mean token count per care history was 12,644, with a maximum of 153,535 tokens and a minimum of 29 tokens.

Basic text preprocessing was applied to the nursing notes. This included removing HTML tags and lemmatization [TurkuNLP neural parser (32)]. Lemmatization is a task in which the goal is to define a base or dictionary form (lemma) for a given word. Next, Finnish stopwords defined in nltk.stopwords (33) were removed, and common measurement indicators, such as “cm” and “kg,” were removed. We did not remove the word “ei” (meaning “no”), which is considered a stopword, to retain possible phrases consisting of a negation. Finally, the datasets were transformed to token count vector format with Scikit-learn (34) package's CountVectorizer with default parameters, except ngram_range was set to uni and bigrams, max_df to 0.8 (ignore tokens that have a document frequency higher than the given threshold), min_df to 5 (ignore tokens that have a document frequency strictly lower than the given threshold), and max_features to 2000. The resulting patient-per-token matrix consisted of 2,000 columns. Each element in each column represents the count of a token or bigram (a textual unit consisting of two adjacent tokens) in a patient's nursing notes. Such a BoW representation was chosen because long-term dependencies are difficult to learn from long text due to minimal layer weight changes in models leveraging recurrence (35). We limited the BoW representation to only include the 2,000 most frequent terms as features to avoid putting too much weight on rare words. This number of features has previously been found to perform well in document classification tasks (36).

### 2.3 Model evaluation

The model evaluation aimed to find the best-performing model architecture and training data. We tested three well-known and widely used ML models for the classification task: NN, RF, and LR. In a classification task, a model is evaluated by its capability to predict the class of unseen data points for which the true label is known. The NN model was implemented using the Transformers (37) and Pytorch (38) packages, while the RF and LR models were implemented with Scikit-learn (34). Classification performances were evaluated with three-times repeated stratified fivefold nested cross-validation using 36 different hyperparameter settings for each model in the inner loop. Hyperparameters are parameters used to control the algorithm's learning process.

All models used balanced label weighting in training. Class weights were defined from each training split using the formula number of samples divided by the number of classes times the number of occurrences of the class. The NN was implemented as a three-layer network consisting of two hidden layers and an output layer, with the complete model ranging from 529,000 to 545,000 parameters. For each fold, the NN was trained for a maximum of 500 epochs, with an early stopping patience of 20. The learning rate, batch size, hidden size of the second layer, and dropout rate were tuned. The RF was implemented as a forest of 300 estimators, and the maximum depth of a tree, the number of features for best split, the minimum number of samples required in a leaf node, and the minimum number of samples required to split an internal node were tuned. The LR was implemented as Elastic-Net. For each fold, the LR was trained for a maximum of 500 iterations, and the regularization strength and L1 ratio were tuned.

The average number of tokens in the care histories consisting of nursing notes is around 12,000, with a maximum of about 150,000. The large number of tokens excludes the possibility of using existing pretrained Transformer-based language models such as Bidirectional Encoder Representations from Transformers (BERT). The pretrained BERT model available for Finnish has a token or input limit of 512 (39).

In cross-validation, data are split into non-overlapping folds on the level of patients, where one fold is used in testing and the rest for training the model. Each fold is used once for testing. In nested cross-validation, two cross-validation loops are formed. The inner loop is used for classifier performance optimization, and the outer loop is used to estimate true classification performance (40).

To evaluate the performance of the models between different training data, both datasets were split into training and testing folds according to unique identifiers of the OCP dataset. This results in the test set for each outer fold containing approximately 15 class 1 patients and 115 class 0 patients, and similarly each inner fold contains approximately 12 class 1 patients and 92 class 0 patients. As an exception to the NN, 10% of the training data in both the outer and the inner folds was set aside as a validation set for early stopping. Repeat splits were done using different random seeds, but each repeat used predefined seeds. With this setup, each fold between the datasets was comparable as they contained the same cancer patient unique identifiers. Model performance was always evaluated only by cancer patients as our goal was to predict the need for support of CCPs. Nested cross-validation was used to ensure as unbiased and low variance performance estimation as possible (41). Data splitting was done with the Scikit-learn (34) package's model_selection module's classes StratifiedKFold and StratifiedShuffleSplit. Mean area under the receiver operating characteristics curve (AUC) was used as a performance measure. AUC was chosen as a metric as it is well-suited for unbalanced binary data, and generally, AUC can be interpreted as the probability that a randomly chosen positive data point is ranked higher than a randomly chosen negative one (42). In addition, AUC gives a good overall estimate of the classification performance without specifying whether the cost of false-positive predictions or false-negative predictions is optimized. The probabilities of models being better than others were computed with Bayesian correlated *t*-tests implemented in Baycomp class CorrelatedTTest (43), and credibility intervals (CIs) and Bayes factors were computed with R (version 4.2.2) using bayestestR (version 0.16.0) (44). Models were considered equal if their AUC differed by less than 0.01 with more than 5% probability. This is called the region of practical equivalence (ROPE). CIs are a Bayesian statistical concept that describes the uncertainty related to an unknown parameter (44). In addition to ROPE, if 95% of the posterior distribution values did not favor one model being better, no superiority conclusion was made. As statistical analysis is done in the Bayesian domain, the relative evidence of the hypothesis is assessed with the Bayes factor instead of the effect size (45). The Bayes factors were computed against the null hypothesis that the difference was within the range of (−∞ to −0.01). Bayes factors in the range of 0.1–0.3 can be interpreted as substantial evidence for the null hypothesis, and 0.01–0.03 as very strong evidence for the null hypothesis (46).

## 3 Results

**Figure 2:**
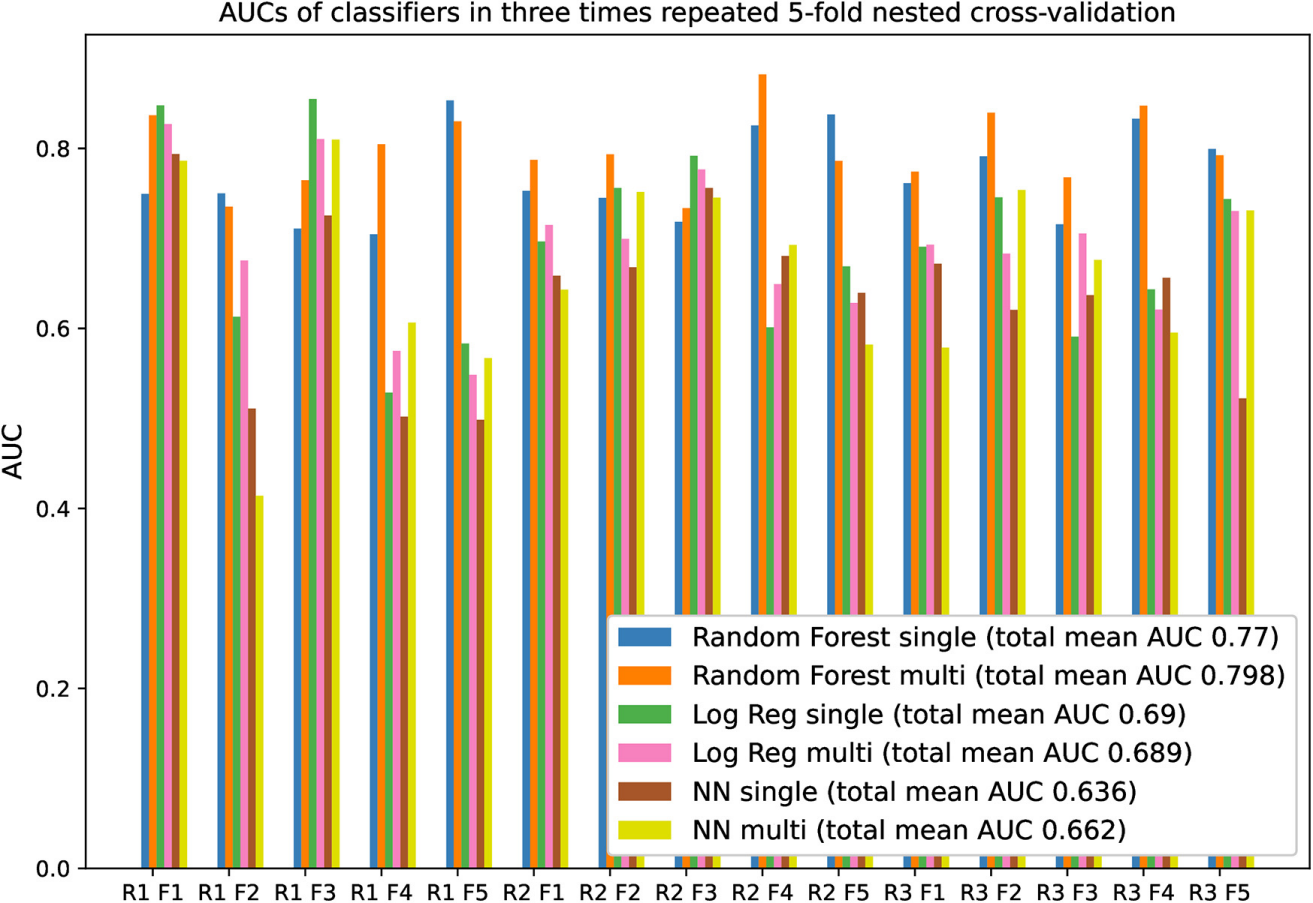
AUCs of classifiers in three-times repeated fivefold nested cross-validation. “R” is an abbreviation of repeat, and “F” is an abbreviation of the fold. The legend suffixes “single” refer to models trained on OCP data, and “multi” to models trained on the whole data. Performance fluctuates between the folds because of the small test set size.

**Figure 3:**
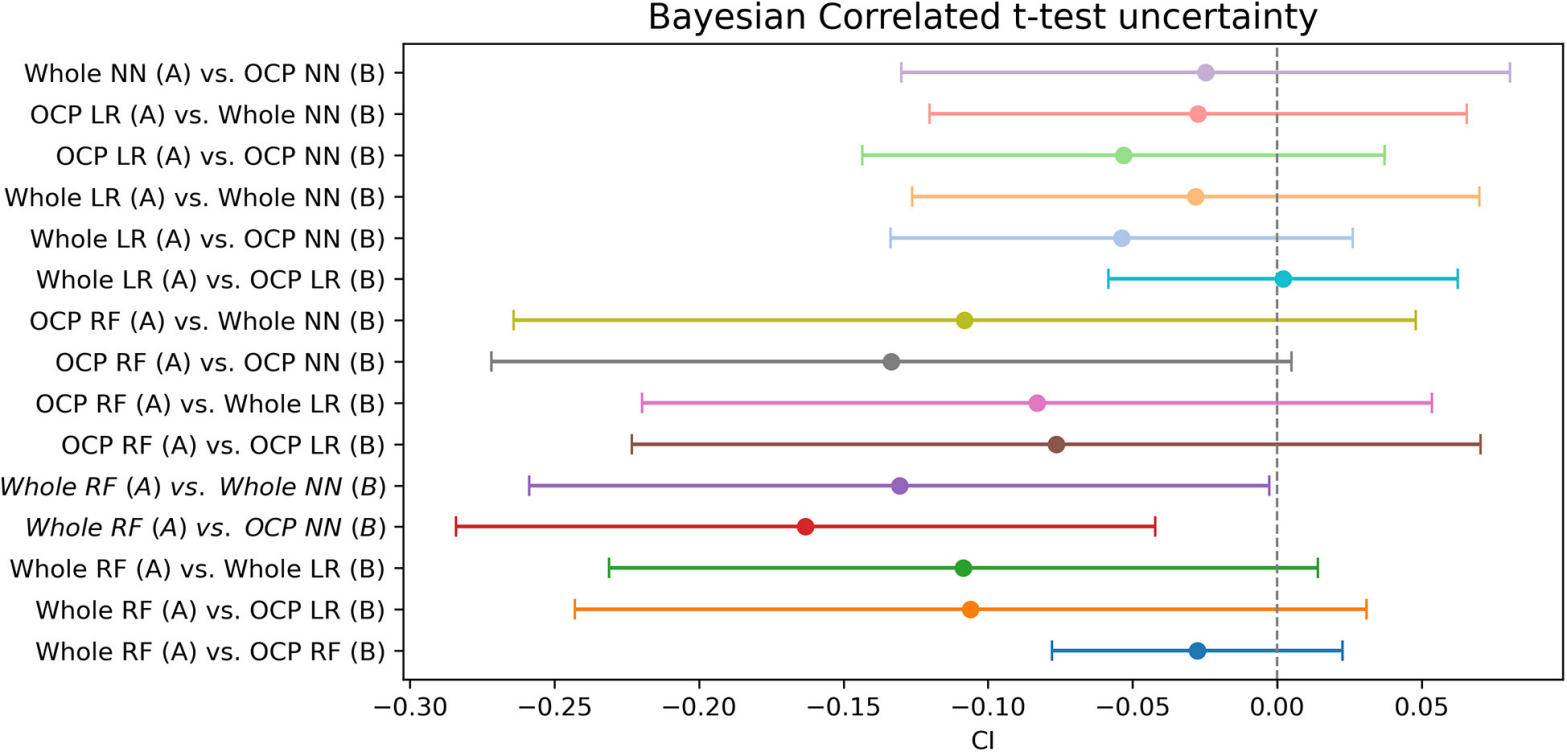
Bayesian t-test uncertainty. The range contains the 95% most probable effect values.

The RF model trained on the whole dataset seems most reliably the best when assessing mean AUC and Bayesian correlated *t*-test results (Table 1). In repeated fivefold nested cross-validation, RF performed with a mean of 0.789 AUC (Figure 2). The RF model trained on the whole dataset was better than both NN models with reasonable credibility (Figure 3), *p* < 0.05 of ROPE and substantial evidence based on the Bayes factor (Table 1). No correction was made due to multiple hypothesis testing, as ROPE is used to assess the credibility of the null values (47). The RF trained with the whole dataset may better represent the true distribution of patients needing support, or the larger training set may introduce regularization against overfitting.

**Table 1:** Bayesian correlated t-test results.

Comparison	A	ROPE	B	CI low	CI high	Bayes factor
Whole RF (A) vs. OCP RF (B)	0.7785	0.1602	0.0612	−0.0779	0.0226	1.4738
Whole RF (A) vs. OCP LR (B)	0.9253	0.0309	0.0438	−0.2430	0.0310	0.4242
Whole RF (A) vs. Whole LR (B)	0.9478	0.0243	0.0278	−0.2311	0.0141	0.2887
*Whole RF* (A) *vs. OCP NN* (B)	*0*.*9915*	*0*.*0043*	*0*.*0042*	*−0.2840*	*−0.0422*	*0*.*0439*
*Whole RF* (A) *vs. Whole NN* (B)	*0*.*9728*	*0*.*0127*	*0*.*0145*	*−0.2586*	*−0.0026*	*0*.*1366*
OCP RF (A) vs. OCP LR (B)	0.8362	0.0576	0.1062	−0.2231	0.0703	0.9957
OCP RF (A) vs. Whole LR (B)	0.8559	0.0548	0.0893	−0.2196	0.0535	0.8773
OCP RF (A) vs. OCP NN (B)	0.9617	0.0166	0.0217	−0.2717	0.0049	0.1951
OCP RF (A) vs. Whole NN (B)	0.8992	0.0364	0.0643	−0.2642	0.0479	0.5738
Whole LR (A) vs. OCP LR (B)	0.3482	0.2717	0.3801	−0.0583	0.0624	9.5247
Whole LR (A) vs. OCP NN (B)	0.8690	0.0764	0.0546	−0.1337	0.0261	0.7928
Whole LR (A) vs. Whole NN (B)	0.6417	0.1417	0.2166	−0.1262	0.0699	2.9146
OCP LR (A) vs. OCP NN (B)	0.8446	0.0807	0.0746	−0.1435	0.0371	0.9676
OCP LR (A) vs. Whole NN (B)	0.6596	0.1439	0.1965	−0.1203	0.0656	2.6315
Whole NN (A) vs. OCP NN (B)	0.6257	0.1361	0.2382	−0.1300	0.0806	3.0618

Columns A and B are probabilities of one model being better than another, where “A” is the first model in a row and “B” is the second. Comparisons considered reliable are marked in italics. Many other comparisons had a high probability that one model was better, but the credibility intervals were too wide to draw any conclusions. Bayesian factors for comparisons considered reliable indicate substantial evidence for model “A” being better.

## 4 Discussion

Cancer patients who had booked outpatient appointments at the mental health–related care units can be recognized from the content in their previous nursing notes by other specialties with good AUC. Most credibly, the best text classification model was based on the RF method. This study demonstrates that the task can be predicted by ML methods. There was not strong enough evidence to claim that using diabetes patients significantly increases performance universally. The most substantial evidence that using the whole dataset increases classification performance came from the Bayesian *T*-test, which compared the RF method trained on the whole dataset with the NN method trained only on cancer patients.

In previous studies, the psychosocial condition of CCPs has been evaluated by questionnaires that demand additional employee resources and manual labor (13, 14). However, the nursing notes written during the patient's hospital journey contain detailed information about the patient's wellbeing and behavior. Nurses are required to document each shift and outpatient contact. This allows the utilization of pre-existing material and possible cost savings. The present study shows the potential for finding signals of distress, depression, maladaptation, and lack of support from guardians and friends. This information is valuable in pointing out the patients and survivors who should, in a timely manner, be referred to specialized support in healthcare settings that do not have systematic psychosocial services provided to childhood and adolescent cancer patients, also in their post-treatment phase.

In future work, we plan to combine the nursing notes with other data from various sources to train a multimodal model for this prediction task. We will have the possibility to validate the method further when we start gathering patient-reported outcome data (PROM) from our cancer patients. The possibility of starting piloting and later routinely implementing the computer-assisted gathering of PROM via PROMIS-CAT (48) has recently been accepted as a research project at our unit. In addition, we aim to explore other solutions for handling the challenges of multidocument classification with word counts that dramatically exceed the limits of currently available pretrained language models like BERT (39) for Finnish. Recently, several Transformer-based architectures (49), such as Linear Transformer (50) and Longformer (51), have been applied to long-text classification problems, and we aim to leverage an architecture from this domain by either pretraining one from scratch ourselves or continuing pretraining of a multilingual variant.

### 4.1 Study limitations

We defined the need for support as an outpatient appointment reservation to a mental health–related care unit. The link between the need for support and reservation in mental health–related care units has not been validated. However, we argue that regardless of the validation, healthcare professionals have seen indicators of the need for support. As patients visit our pediatric hematology-oncology outpatient clinic regularly (at least annually), both during and after treatment, we maintain contact with them up to the age of 18 years and thereafter via the digital long-term follow-up tool. Thus, we make referrals to the mental health team whenever a problem is recognized during routine visits with the oncologist or in adult survivors through the quality-of-life questionnaires sent via the long-term follow-up clinic.

Using a single university hospital dataset might be a limitation, but in Finland, we know that only one out of five centers treating CCPs has a psychologist included in their own staff. All others have the same situation as us, where the clinicians seeing the patients at follow-up visits should themselves be able to recognize the need of patients for psychosocial support and remit them accordingly. On the other hand, using a dataset in one hospital only could better exclude potential biases in the dataset as the documentation practices are similarly governed by the hospital administration, and the journal structure for nursing notes is similar. In addition to this, access to mental health services within one hospital follows equal patterns.

The evaluation of the usefulness of models in a clinical setting remains to be seen, but the results are promising. The final sample for evaluation was relatively small; thus, more data would be needed for a more robust evaluation and probability estimations.

Transformer architecture–based pretrained models have become the preferred choice for natural language processing tasks as they are often the most performant ones (52). We could not leverage Transformer models as no pretrained models suitable for Finnish long text are available.

## 5 Conclusions

Using the predictions provided by the tested classification models may help healthcare professionals identify patients with similar health trajectories and documentation to those who have reservations in mental health–related care units and, thus, better identify those patients who would benefit from (early) mental health–related consultations. Overall, this study indicates that it is possible to find indications for the likely need for psychological support in the future by analyzing the documented content in nursing notes. However, further research is needed to better understand the intrinsic and extrinsic utility of the tested models.

## Data Availability

The data in this study are available within Auria Clinical Informatics with a grant from the Hospital District of Southwest Finland. Accredited researchers can make access applications. For more details, please see https://www.auria.fi/tietopalvelu/en/tutkijalle/index.html section for permissions on research based on the secondary use of patient records. Requests to access the datasets should be directed to tutkimuksentietopalvelut@varha.fi.

## References

[1] Lam CG Howard SC Bouffet E Pritchard-Jones K. Science and health for all children with cancer. Science. (2019) 363(6432):1182–6. 10.1126/science.aaw4892 30872518

[2] Winther JF Kremer L. Long-term follow-up care needed for children surviving cancer: still a long way to go. Lancet Oncol. (2018) 19(12):1546–8. 10.1016/S1470-2045(18)30657-0 30416075

[3] Wiener L Kazak AE Noll RB Patenaude AF Kupst MJ. Standards for the psychosocial care of children with cancer and their families: an introduction to the special issue. Pediatr Blood Cancer. (2015) 62(Suppl 5):S419–24. 10.1002/pbc.25675 26397836 PMC6397048

[4] Loonen JJ Blijlevens NM Prins J Dona DJ Den Hartogh J Senden T Cancer survivorship care: person centered care in a multidisciplinary shared care model. Int J Integr Care. (2018) 18(1):4. 10.5334/ijic.3046 29588641 PMC5854087

[5] Weinstein AG Henrich CC Armstrong GT Stratton KL King TZ Leisenring WM Roles of positive psychological outcomes in future health perception and mental health problems: a report from the childhood cancer survivor study. Psychooncology. (2018) 27(12):2754–60. 10.1002/pon.4881 30189119 PMC6452629

[6] Brinkman TM Recklitis CJ Michel G Grootenhuis MA Klosky JL. Psychological symptoms, social outcomes, socioeconomic attainment, and health behaviors among survivors of childhood cancer: current state of the literature. J Clin Oncol. (2018) 36(21):2190–7. 10.1200/JCO.2017.76.5552 29874134 PMC6053297

[7] Söllner W DeVries A Steixner E Lukas P Sprinzl G Rumpold G How successful are oncologists in identifying patient distress, perceived social support, and need for psychosocial counselling? Br J Cancer. (2001) 84(2):179–85. 10.1054/bjoc.2000.1545 11161373 PMC2363697

[8] Bruce M. A systematic and conceptual review of posttraumatic stress in childhood cancer survivors and their parents. Clin Psychol Rev. (2006) 26(3):233–56. 10.1016/j.cpr.2005.10.002 16412542

[9] Crochet E Tyc VL Wang M Srivastava DK Van Sickle K Nathan PC Posttraumatic stress as a contributor to behavioral health outcomes and healthcare utilization in adult survivors of childhood cancer: a report from the childhood cancer survivor study. J Cancer Surviv. (2019) 13(6):981–92. 10.1007/s11764-019-00822-5 31691097 PMC6883135

[10] Frederiksen LE Mader L Feychting M Mogensen H Madanat-Harjuoja L Malila N Surviving childhood cancer: a systematic review of studies on risk and determinants of adverse socioeconomic outcomes. Int J Cancer. (2019) 144(8):1796–823. 10.1002/ijc.31789 30098012

[11] Wallace WH Blacklay A Eiser C Davies H Hawkins M Levitt GA Developing strategies for long term follow up of survivors of childhood cancer. Br Med J. (2001) 323(7307):271–4. 10.1136/bmj.323.7307.271 11485960 PMC1120887

[12] Eiser C Absolom K Greenfield D Glaser A Horne B Waite H Follow-up after childhood cancer: evaluation of a three-level model. Eur J Cancer. (2006) 42(18):3186–90. 10.1016/j.ejca.2006.08.001 16989995

[13] Nofech-Mozes J Hancock K Chung J Barrera M. Psychosocial difficulties identified by health care providers as they predict pain-related quality of life in children with cancer. Support Care Cancer. (2020) 28(7):3459–66. 10.1007/s00520-019-05195-0 31802251

[14] Okado Y Howard Sharp KM Tillery R Long AM Phipps S. Profiles of dispositional expectancies and affectivity predict later psychosocial functioning in children and adolescents with cancer. J Pediatr Psychol. (2016) 41(3):298–308. 10.1093/jpepsy/jsv096 26476282 PMC5013836

[15] International Guideline Harmonization Group. International guideline harmonization group. Available online at: https://www.ighg.org/ (Accessed October 15, 2024).

[16] Rajala S Järvelä LS Huurre A Grönroos M Rautava P Lähteenmäki PM. Use of electronic patient data storage for evaluating and setting the risk category of late effects in childhood cancer survivors. Pediatr Blood Cancer. (2020) 67(11):e28678. 10.1002/pbc.28678 32860665

[17] Kazak AE Hwang W-T Chen FF Askins MA Carlson O Argueta-Ortiz F Screening for family psychosocial risk in pediatric cancer: validation of the psychosocial assessment tool (PAT) version 3. J Pediatr Psychol. (2018) 43(7):737–48. 10.1093/jpepsy/jsy012 29509908

[18] Devine KA Christen S Mulder RL Brown MC Ingerski LM Mader L Recommendations for the surveillance of education and employment outcomes in survivors of childhood, adolescent, and young adult cancer: a report from the international late effects of childhood cancer guideline harmonization group. Cancer. (2022) 128(13):2405–19. 10.1002/cncr.34215 35435238 PMC9321726

[19] Marchak JG Christen S Mulder RL Baust K Blom JMC Brinkman TM Recommendations for the surveillance of mental health problems in childhood, adolescent, and young adult cancer survivors: a report from the international late effects of childhood cancer guideline harmonization group. Lancet Oncol. (2022) 23(4):e184–96. 10.1016/S1470-2045(21)00750-6 35358467 PMC9639707

[20] Huang T Ngan C-K Cheung YT Marcotte M Cabrera B. A hybrid deep learning-based feature selection approach for supporting early detection of long-term behavioral outcomes in survivors of cancer: cross-sectional study. JMIR Bioinform Biotech. (2025) 6:e65001. 10.2196/65001 PMC11950700 40080820

[21] Sheikhalishahi S Miotto R Dudley JT Lavelli A Rinaldi F Osmani V. Natural language processing of clinical notes on chronic diseases: systematic review. JMIR Med Inform. (2019) 7(2):e12239. 10.2196/12239 31066697 PMC6528438

[22] Zeinali N Youn N Albashayreh A Fan W Gilbertson White S. Machine learning approaches to predict symptoms in people with cancer: systematic review. JMIR Cancer. (2024) 10:e52322. 10.2196/52322 38502171 PMC10988375

[23] McRoy S Rastegar-Mojarad M Wang Y Ruddy KJ Haddad TC Liu H. Assessing unmet information needs of breast cancer survivors: exploratory study of online health forums using text classification and retrieval. JMIR Cancer. (2018) 4(1):e10. 10.2196/cancer.9050 29764801 PMC5974460

[24] Lu S-C Xu C Nguyen CH Geng Y Pfob A Sidey-Gibbons C. Machine learning-based short-term mortality prediction models for patients with cancer using electronic health record data: systematic review and critical appraisal. JMIR Med Inform. (2022) 10(3):e33182. 10.2196/33182 35285816 PMC8961346

[25] Masukawa K Aoyama M Yokota S Nakamura J Ishida R Nakayama M Machine learning models to detect social distress, spiritual pain, and severe physical psychological symptoms in terminally ill patients with cancer from unstructured text data in electronic medical records. Palliat Med. (2022) 36(8):1207–16. 10.1177/02692163221105595 35773973

[26] Nunez J-J Leung B Ho C Ng RT Bates AT. Predicting which patients with cancer will see a psychiatrist or counsellor from their initial oncology consultation document using natural language processing. Commun Med (Lond). (2024) 4(1):69. 10.1038/s43856-024-00495-x 38589545 PMC11001970

[27] Korach ZT Cato KD Collins SA Kang MJ Knaplund C Dykes PC Unsupervised machine learning of topics documented by nurses about hospitalized patients prior to a rapid-response event. Appl Clin Inform. (2019) 10(5):952–63. 10.1055/s-0039-3401814 31853936 PMC6920051

[28] Moen H Hakala K Mehryary F Peltonen L-M Salakoski T Ginter F “Detecting mentions of pain and acute confusion in Finnish clinical text”. In: Cohen KB, Demner-Fushman D, Ananiadou S, Tsujii J, editors. BioNLP 2017. Stroudsburg, PA: Association for Computational Linguistics (2017). p. 365–72.

[29] Liukas T Rosio R Peltonen L-M. Towards automated risk prediction of persistent pain: exploring psychosocial factors from electronic health records after breast cancer surgery. Nurs Open. (2023) 10(5):3399–405. 10.1002/nop2.1594 36598880 PMC10077400

[30] Reunamo A. Code for analysis used in the article “Supervised machine learning applied nursing notes for identifying childhood cancer patients’ need for psychosocial support” (2025). Available online at: https://github.com/rakseli/childhood-cancer-mh-prediction (Accessed January 30, 2023). 10.3389/fdgth.2025.1585309 PMC12367647 40851639

[31] Collins GS Reitsma JB Altman DG Moons KGM. Transparent reporting of a multivariable prediction model for individual prognosis or diagnosis (TRIPOD): the TRIPOD statement. Ann Intern Med. (2015) 162(1):55–63. 10.7326/M14-0697 25560714

[32] Kanerva J Ginter F Salakoski T. Universal Lemmatizer: a sequence-to-sequence model for lemmatizing Universal Dependencies treebanks. Nat Lang Eng. (2021) 27(5):545–74. 10.1017/S1351324920000224

[33] Bird S Klein E Loper E. Natural Language Processing with Python: Analyzing Text with the Natural Language Toolkit. 1st ed. Beijing, China: O’Reilly Media (2009).

[34] Pedregosa F Varoquaux G Gramfort A Michel V Thirion B Grisel O Scikit-learn: machine learning in Python. J Mach Learn Res. (2011) 12:2825–30. 10.5555/1953048.2078195

[35] Hochreiter S. The vanishing gradient problem during learning recurrent neural nets and problem solutions. Int J Uncertain Fuzziness Knowl Based Syst. (1998) 6(2):107–16. 10.1142/S0218488598000094

[36] HaCohen-Kerner Y Miller D Yigal Y. The influence of preprocessing on text classification using a bag-of-words representation. PLoS One. (2020) 15(5):e0232525. 10.1371/journal.pone.0232525 32357164 PMC7194364

[37] Wolf T Debut L Sanh V Chaumond J Delangue C Moi A Transformers: state-of-the-art natural language processing. Proceedings of the 2020 Conference on Empirical Methods in Natural Language Processing: System Demonstrations. Stroudsburg, PA: Association for Computational Linguistics (2020). p. 38–45.

[38] Paszke A Gross S Massa F Lerer A Bradbury J Chanan G PyTorch: an imperative style, high-performance deep learning library. In: Wallach H, Larochelle H, Beygelzimer A, d'Alché-Buc F, Fox E, Garnett R, editors. Proceedings of the 33rd International Conference on Neural Information Processing Systems. Red Hook, NY: Curran Associates Inc. (2019). p. 721.

[39] Virtanen A Kanerva J Ilo R Luoma J Luotolahti J Salakoski T Multilingual is not enough: BERT for Finnish. arXiv [preprint]. *arXiv:1912.07076* (2019). Available online at: 10.48550/arXiv.1912.07076 (Accessed January 24, 2025).

[40] Varma S Simon R. Bias in error estimation when using cross-validation for model selection. BMC Bioinform. (2006) 7:91. 10.1186/1471-2105-7-91 PMC1397873 16504092

[41] Cawley GC Talbot NL. On over-fitting in model selection and subsequent selection bias in performance evaluation. J Mach Learn Res. (2010) 11:2079–107. 10.5555/1756006.1859921

[42] Fawcett T. An introduction to ROC analysis. Pattern Recognit Lett. (2006) 27(8):861–74. 10.1016/j.patrec.2005.10.010

[43] Benavoli A Corani G Demšar J Zaffalon M. Time for a change: a tutorial for comparing multiple classifiers through Bayesian analysis. J Mach. (2017) 18(77):1–36. 10.5555/3122009.3176821

[44] Makowski D Ben-Shachar M Lüdecke D. Bayestestr: describing effects and their uncertainty, existence and significance within the Bayesian framework. JOSS. (2019) 4(40):1541. 10.21105/joss.01541

[45] Morey RD Rouder JN. Bayes factor approaches for testing interval null hypotheses. Psychol Methods. (2011) 16(4):406–19. 10.1037/a0024377 21787084

[46] Wetzels R Matzke D Lee MD Rouder JN Iverson GJ Wagenmakers E-J. Statistical evidence in experimental psychology: an empirical comparison using 855 *t* tests. Perspect Psychol Sci. (2011) 6(3):291–8. 10.1177/1745691611406923 26168519

[47] Kruschke JK. Bayesian estimation supersedes the *t* test. J Exp Psychol Gen. (2013) 142(2):573–603. 10.1037/a0029146 22774788

[48] HealthMeasures, Northwestern University’s Feinberg School of Medicine. HealthMeasures. Available online at: https://www.healthmeasures.net/ (Accessed October 15, 2024).

[49] Vaswani A Shazeer N Parmar N Uszkoreit J Jones L Gomez AN Attention is all you need. arXiv [preprint]. *arXiv:1706.03762* (2017). Available online at: 10.48550/arXiv.1706.03762 (Accessed January 24, 2025).

[50] Katharopoulos A Vyas A Pappas N Fleuret F. Transformers are RNNs: fast autoregressive transformers with linear attention. arXiv [preprint]. *arXiv:2006.16236* (2020). Available online at: 10.48550/arXiv.2006.16236 (Accessed January 24, 2025).

[51] Beltagy I Peters ME Cohan A. Longformer: the long-document transformer. arXiv [preprint]. *arXiv:2004.05150* (2020). Available online at: 10.48550/arXiv.2004.05150 (Accessed January 24, 2025).

[52] Lin T Wang Y Liu X Qiu X. A survey of transformers. AI Open. (2022) 3:111–32. 10.1016/j.aiopen.2022.10.001

